# Editorial: System dynamics modeling in public health: implementations and implications

**DOI:** 10.3389/fpubh.2025.1713419

**Published:** 2025-11-10

**Authors:** Kimberly Jinnett, David W. Lounsbury, Nasim S. Sabounchi, Reda Lebcir, Claire F. Brereton

**Affiliations:** 1 Institute for Health & Aging, Department of Social & Behavioral Sciences, UCSF School of Nursing, University of California, San Francisco, San Francisco, CA, United States; 2 Epidemiology and Population Health, Albert Einstein College of Medicine, New York, NY, United States; 3 CUNY Graduate School of Public Health & Health Policy, New York, NY, United States; 4 Hertfordshire Business School, Department of Business Analytics and Systems, University of Hertfordshire, Hatfield, United Kingdom; 5 UQ Centre for Clinical Research, The University of Queensland, Brisbane, QLD, Australia

**Keywords:** system dynamics, implementation, modeling, community-based, group model building

Public health challenges are inherently complex. Health outcomes are not the result of simple, linear chains of cause and effect, but emerge from the dynamic interplay of health behaviors, environmental and socioeconomic conditions, service delivery infrastructures, policy decisions, financing of the healthcare sector, and disease progression over time. Understanding and intervening in such systems requires methodologies that can embrace this complexity. System Dynamics (SD), with its focus on feedback loops, time delays, and non-linear relationships, provides a powerful lens for this purpose. In addition, SD offers policy makers with a “virtual laboratory” to explore how systems behave under alternative scenarios, predict potential responses to interventions, and translate complex evidence into actionable insights.

The goal of this Research Topic is to highlight innovative approaches to SD modeling in public health with an emphasis on implementations and implications. The featured articles in this Research Topic demonstrate the utility of system dynamics as a robust, mixed-methods approach to fostering deeper understanding of some of today's most pressing health issues and insights about their potential solutions from authors across the globe including Australia, Brazil, Cameroon, Jamaica, Kenya, Mexico, South Africa, United Arab Emirates, United Kingdom, and United States.

The Research Topic covers a wide spectrum of public health domains, illustrating the versatility of systems approaches. Articles explore the drivers of maternal health disparities among Black women in Texas (Brown et al.), model the dynamics of gender-based family violence in Mexico (Torres Angeles et al.) and assess the long-term predictive validity of infectious disease forecasts for the COVID-19 pandemic in the UK (Bowie and Friston). Further, the Research Topic examines the design of community-clinical linkages in Brooklyn to address social needs (Toney et al.), the complex factors contributing to alcohol-involved sexual violence on college campuses (Moore et al.), and the intersection of community climate resilience and health in the Global South (Morais et al.). A foundational perspective by Silburn makes the overarching case for applying systems thinking to public health policy.

A central theme emerging from this Research Topic is the breadth of modeling paradigms, from expert-driven quantitative simulations to deeply participatory qualitative mapping. On one end of the spectrum, Torres Angeles et al. use a compartmental model with nonlinear ordinary differential equations while Bowie and Friston apply Dynamic Causal Modeling. These approaches synthesize large qualitative and quantitative datasets to yield qualitative insights and simulated results that inform comparative effectiveness research and multi-dimensional, multi-leveled policy analysis.

On the other end, and a standout feature of this Research Topic, is the strong emphasis on Community-Based System Dynamics (CBSD) and Group Model Building (GMB). Brown et al., Morais et al., and Toney et al. place stakeholder and community engagement at the core of their procedures. Using methods grounded in co-learning and co-production, they build causal loop diagrams (CLDs) that reveal core drivers of complex problems that capture the lived experiences and mental models of those most affecting community health and quality of health service delivery. This participatory approach not only enriches the model's structure but also fosters shared understanding and builds the trust necessary for collective action. Bridging these two paradigms, Moore et al. demonstrate a powerful hybrid approach, translating collaboratively developed qualitative diagrams into a formal stock-and-flow structure that provides a rigorous, systems-based theory of change ready for future quantification. There is no artificial qualitative-quantitative divide in these approaches. The value of community data input to a quantitative model and community feedback on the scenarios and findings of the model are all a part of CBSD.

Despite their methodological diversity, the articles are united by a common purpose: to move from insight to action. The models are not academic exercises; they are decision-support tools designed to identify high-leverage points for intervention. The models of violence by Torres Angeles et al. and Moore et al. reveal how intervention mechanisms, such as rehabilitation and bystander training, can disrupt harmful reinforcing cycles. The community-built models from Brown et al. and Toney et al. are explicitly designed to support more equitable, community-informed health programs and policy.

This Research Topic offers important implications for the broader field of systems science. First, it affirms the field's unique capacity to tackle complex problems where social, behavioral, and biological factors are tightly interwoven. Second, it highlights a mature and healthy methodological pluralism. The successful integration of “hard” quantitative simulation with “soft” participatory modeling demonstrates that the field is evolving to meet the demands of complex social challenges where human behavior and stakeholder buy-in are as critical as empirical data. The lessons learned from implementing GMB in a trauma-informed way (Brown et al.) or blending CBSD with design thinking (Toney et al.) represent significant practical contributions.

We hope readers will agree that the articles in this Research Topic powerfully illustrate the value of system dynamics modeling in public health. They showcase a field that is methodologically innovative, deeply engaged with communities, and committed to producing actionable knowledge. By embracing dynamic complexity, these researchers provide not just a clearer understanding of the problems we face, but a structured and hopeful path toward designing more effective and equitable solutions.

